# The Impact of Transoral Robotic Surgery on Erectile Dysfunction and Lower Urinary Tract Symptoms in Male Patients with Moderate-to-Severe Obstructive Sleep Apnea

**DOI:** 10.3390/healthcare10091633

**Published:** 2022-08-26

**Authors:** Chih-Kai Peng, Chien-Han Tsao, Wen-Wei Sung, Shao-Chuan Wang, Wen-Jung Chen, Tzuo-Yi Hsieh, Min-Hsin Yang, Tsung-Hsien Lee, Sung-Lang Chen

**Affiliations:** 1 Department of Urology, Chung Shan Medical University Hospital, Taichung 402, Taiwan; 2 Institute of Medicine, Chung Shan Medical University, Taichung 402, Taiwan; 3 Department of Otolaryngology, Chung Shan Medical University Hospital, Taichung 402, Taiwan; 4 School of Medicine, Chung Shan Medical University, Taichung 402, Taiwan; 5 Department of Obstetrics and Gynecology, Chung Shan Medical University Hospital, Taichung 402, Taiwan

**Keywords:** obstructive sleep apnea, lower urinary tract symptoms, erectile dysfunction, transoral robotic surgery

## Abstract

Obstructive sleep apnea (OSA), lower urinary tract symptoms (LUTS), and erectile dysfunction (ED) are chronic conditions that seriously affect middle-aged men. This study aimed to evaluate the changes in the presence of these conditions after transoral robotic surgery (TORS) for OSA. This prospective observational study recruited 48 men with moderate-to-severe OSA (mean age 40.6 ± 8.1 years) who underwent TORS from October 2019 to November 2021 at a tertiary center. Baseline polysomnographic parameters, Epworth Sleepiness Scale (ESS), and demographic characteristics were measured. The evaluations of LUTS and ED were based on self-administered International Prostate Symptom Score (IPSS) and International Index of Erectile Function (IIEF-5) questionnaires, respectively, before TORS. The treatment outcomes were assessed three months postoperatively in the patients undergoing TORS due to moderate-to-severe OSA. There was significant Apnea-Hypopnea Index (AHI) reduction from 53.10 ± 25.77 to 31.66 ± 20.34 three months after undergoing TORS (*p* < 0.001). There was also a significant decrease in the total IPSS score (5.06 ± 5.42 at baseline to 2.98 ± 2.71 at three months postoperatively, *p* = 0.001), the storage domain, and the voiding domain (*p* < 0.05). The ED also improved significantly, as seen in the IIEF score (20.98 ± 3.32 to 22.17± 3.60, *p* = 0.007). The reduction of AHI was associated with changes in body weight and the lowest oxygen saturation (SpO_2_) levels during sleep (rho = 0.395, *p* = 0.005; rho = 0.526, *p* < 0.001, respectively). However, the reduction in AHI was not significantly associated with improvement in IPSS or IIEF scores (*p* > 0.05). For men with moderate-to-severe OSA, TORS can significantly improve the polysomnography parameters, sleep-related questionnaire scores, and quality of life, and alleviate ED and LUTS. AHI reduction is not a crucial factor for ED and LUTS improvement after TORS for OSA, especially in ED.

## 1. Introduction

Obstructive sleep apnea (OSA) is a common chronic disease in middle-aged men with significant comorbidity, including cardiovascular dysfunction, diabetes mellitus, mental health diseases, and urological problems [1,2]. A person with OSA usually experiences sleep fragmentation and intermittent hypoxia while sleeping. Intermittent hypoxemia was also thought to be a crucial factor that resulted in OSA-related comorbidities with the mechanism of ischemia-reperfusion injury [1].

Erectile dysfunction (ED) is a type of sexual dysfunction defined as the inability to achieve and maintain an erection strong enough to reach sexual satisfaction [3]. Guilleminault et al. defined the association between OSA and ED for the first time and found the prevalence of ED to be 48% in patients with severe OSA [4]. Lower urinary tract symptoms (LUTS) are also a common urological problem in men; they include voiding or storage symptoms. A high prevalence of nocturia in patients with OSA has been reported, and nocturia is also thought to be a multiple etiology symptom in several studies [5]. Lombardi et al. advocated the relationship between OSA severity and the degree of sympathetic overactivity [6]. McVary also proposed that increased sympathetic tone was the common link between ED and LUTS [7]. The exact mechanism of ED and LUTS in patients with OSA remains unclear, but it is recognized to be multifactorial, such as endothelial dysfunction, increased sympathetic tone, hypoxia, and a reduction in nitric oxide (NO) formation [8]. Furthermore, few studies have evaluated the presence of both ED and LUTS in patients with OSA or have shown the changes in these conditions and quality of life (QOL) after OSA treatment [9].

Continuous positive airway pressure (CPAP) treatment is one of the effective treatments for OSA. Research has demonstrated that CPAP treatment for OSA could lessen the severity of ED and LUTS [10,11]. However, poor adherence to CPAP precludes its effectiveness. Multiple alternative treatment options are being advocated. Upper airway surgery is the treatment of choice in selected patients who have refused or cannot tolerate CPAP. Over the years, the most-performed surgical procedure for OSA has been uvulopalatopharyngoplasty (UPPP), which aims to increase the retropalatal lumen and reduce the collapsibility of the pharynx by resection of the free edge of the uvula and soft palate, often in combination with a tonsillectomy [12,13]. While UPPP has been shown to be beneficial, the measure of the outcomes in the papers was controversial [14]. Furthermore, this procedure was associated with a high incidence of unfavorable postoperative complications and morbidities.

Transoral robotic surgery (TORS) was first described by O’Malley et al. in 2006 to treat upper aerodigestive tract neoplasms [15]. Vicini et al. published a preliminary report on TORS for volumetric reduction of the tongue base in their multilevel surgical management strategy for treating OSA in 2010 [16]. TORS for OSA is still a relatively new intervention and it has been found to have encouraging results in the postoperative Epworth Sleepiness Score (ESS) and the Apnea-Hypopnea Index (AHI), the index of evaluation used to determine OSA improvement [17]. However, the efficacies of TORS for improving both ED and LUTS have not yet been investigated.

In the present study, we evaluated the effects of TORS-assisted OSA surgery on LUTS and ED in male patients with moderate-to-severe OSA. We also analyzed the correlations among the improvement of ED, LUTS, and OSA after TORS-assisted OSA surgery.

## 2. Materials and Methods

### 2.1. Patients and Methods

This open prospective study was conducted collaboratively by the Urology Department and the Otolaryngology Department at Chung Shan Medical University Hospital (CSMUH). The study was approved by the Institutional Review Board of Chung Shan Medical University (CS19120 and CS2-21108) and was performed according to the ethical principles of the Good Clinical Practice Guidelines and the principles outlined in the Declaration of Helsinki. Forty-eight males with moderate-to-severe OSA who underwent TORS and met our inclusion criteria were included in the study to evaluate LUTS and ED improvement. As a tertiary referral center for TORS, informed consent is an essential step in helping patients to be aware of consequences of their further treatment decisions. Informed consent was obtained from all the patients before the study. All the consecutively enrolled male OSA patients were over the age of 18 and underwent TORS for OSA between October 2019 and November 2021, which was performed by the same otolaryngologist (Chien-Han Tsao) at CSMUH.

The eligible patients had polysomnography (PSG) with an AHI ≥ 15 per hour of total sleep time and refused or had at least 12-week failed conventional treatment, such as an oral appliance or CPAP. The exclusion criteria were women, younger than eighteen years of age, contraindications for OSA surgery under general anesthesia, chronic use of sleeping pills, or undergoing medical treatment, such as taking α-blockers, 5α-reductase inhibitors, anticholinergics, and phosphodiesterase type 5 inhibitors for ED or LUTS. Patients who had a past history of urogenital surgery, urological cancer, or neurogenic bladder, uncontrolled hypertension, diabetes, renal disorders, or other major medical diseases, such as heart failure, deep vein thrombosis, peripheral vascular disease, or chronic lung diseases were also excluded.

All patients received PSG and completed the International Index of Erectile Function (IIEF-5), International Prostate Symptom Score (IPSS), and Epworth Sleepiness Scale (ESS) questionnaires preoperatively and three months postoperatively. The basic characteristics of the patients, including age, height, body weight (BW), body mass index (BMI), neck circumference, systemic disease, drug use, and smoking history, were recorded.

The primary endpoint of our study was improvements in OSA, LUTS, and ED after TORS. We also aimed to explore the association of improvements among LUTS, ED, and OSA.

### 2.2. Polysomnography

All patients underwent PSG to assess the severity of OSA with AHI before TORS. PSG was performed again three months after surgery at our cooperating sleep center. The PSG with a Rembrandt system (Rembrandt, Medcare, Amsterdam, the Netherlands) was used to record the brain waves, electrocardiography, oxygen saturation (SpO_2_), heart rate, sleep architecture, respiratory movement, and periodic leg movements (PLMs) while sleeping. AHI was calculated to indicate the severity of sleep apnea. AHI is the number of apnea or hypopnea events per hour during sleep. Based on the AHI, the severity of OSA is classified as mild (5 ≤ AHI < 15), moderate (15 ≤ AHI < 30), or severe (AHI ≥ 30) [5,9]. Lowest SpO_2_ was also recorded for the analyses.

### 2.3. Transoral Robotic Surgery (TORS)

TORS combined with UPPP and laterolpharyngoplasty was performed for all patients under general anesthesia. Bilateral tonsils were also removed, and the partial tongue base and redundant pillar tissue were resected to improve the oropharyngeal space [12,18]. All surgeries were performed by the same otolaryngologist (Chien-Han Tsao) who has performed TORS on more than 100 patients. Possible peri- and postoperative adverse events were also recorded.

### 2.4. Questionnaires

All the patients completed the IIEF-5 and IPSS questionnaires preoperatively and three months postoperatively. The patients completing the baseline IIEF-5 and IPSS questionnaires were interviewed by a urologist in person, and the follow-up questionnaires were filled out over the telephone or by using communication software. Pre- and postoperative PSG examinations were conducted, BW and BMI were measured, and the ESS questionnaire was completed at the same time by the sleep laboratory staff.

#### 2.4.1. International Index of Erectile Function (IIEF)

IIEF-5 is used to assess ED. It is composed of five questions, and each item is rated on a 5-point scale, ranging from 0 = very low to 5 = very high. A lower score indicates more severe ED. The total score is 25, and ED was indicated with a score of ≤21. Additionally, the severity of ED is classified as mild (12–21), moderate (8–11), or severe (5–7) [11].

#### 2.4.2. International Prostate Symptom Score (IPSS)

IPSS is used to evaluate LUTS. It is composed of seven questions, and each item is rated on a 5-point scale, ranging from 0 to 5. A higher score indicates more severe urinary symptoms. The seven items were divided into storage symptoms (frequency, urgency, nocturia) and voiding symptoms (incomplete emptying, intermittency, weak stream, straining). The total score is 35, and the severity of LUTS is classified as mild (0–7), moderate (8–19), or severe (20–35) [19].

#### 2.4.3. Epworth Sleepiness Scale (ESS)

ESS is a questionnaire that evaluates the subjective level of daytime sleepiness. It uses a 3-point scale (0 = never to 3 = high probability) to rate the probability of falling asleep in eight situations. Adding the scores of all the questions yields a total score ranging from 0 to 24. A score of 0–9 is considered to be normal; a score > 10 indicates excessive daytime sleepiness and recommendations for medical advice [11,20].

### 2.5. Statistical Analysis

Descriptive statistics of all the variables were performed. Each continuous variable was presented as the mean and standard deviation, and the categorical variable was expressed as number (n) and percentage (%). To evaluate the effect on LUTS and ED after surgical correction of OSA, we calculated the changes in the IPSS and IIEF scores. Differences between the baseline and the postoperative three-month scores were compared with a paired *t*-test. Subgroup analyses were performed using the student’s *t*-test and chi-squared test. Spearman’s correlation was used to measure the strength of association among the changes in IPSS, IIEF, AHI, BW, and lowest SpO_2_. A *p* value < 0.05 was regarded as significant. All statistical analyses were performed using IBM SPSS version 25.

## 3. Results

### 3.1. Baseline Characteristics

The mean age of the study participants was 40.6 ± 8.1 years, and the mean AHI was 53.10 ± 25.77 at baseline. Eleven (22.9%) patients had moderate OSA (15 ≤ AHI < 30) and 37 (77.1%) had severe OSA (AHI ≥ 30). The patients’ comorbidities were listed in Table 1.

### 3.2. Postoperative Changes in the Parameters

After TORS, a significant decrease in BW and BMI was found: from 85.16 ± 17.36 to 82.28 ± 15.73 kg and 28.24 ± 5.38 to 27.28 ± 4.90 kg/m^2^, respectively (*p* < 0.001). The mean ESS score was found to have a significant improvement: from 8.15 ± 4.73 to 6.19 ± 3.42 (*p* = 0.005). The neck circumference and lowest SpO_2_ were more favorable postoperatively (*p* ≤ 0.001). There was significant mean AHI reduction from 53.10 ± 25.77 to 31.66 ± 20.34 postoperatively (*p* < 0.001) (Table 1).

Postoperatively, there was also a significant reduction in nocturia (*p* = 0.047), the IPSS score (*p* = 0.001), the storage symptom score (*p* = 0.002), the voiding symptom score (*p* = 0.011), and QOL in IPSS (*p* = 0.005). The mean erectile function also improved significantly, as seen in the IIEF score (*p* = 0.007) postoperatively (Table 2).

The 48 patients were divided into a younger age group and an older age group (cut-off point was 40 years of age, the median age). The mean age was 47.0 ± 3.7 years in the older age group and 33.0 ± 4.7 years in the younger age group. Significant reduction in BW, AHI, and the IPSS score was observed in both groups. However, only the older age group had an obvious improvement in ESS, QoL-IPSS, and IIEF (*p* < 0.05) (Table 3).

The successful TORS outcome for OSA was defined as a reduction of at least 50% in preoperative AHI or a postoperative AHI < 20 per hour [5,21]. Our success rate for TORS-assisted OSA surgery was 45.8% (22/48). Regardless of whether or not the AHI improvement fulfilled the criterion for success, both the successful and unsuccessful groups demonstrated a statistically significant reduction in BW, neck circumference, AHI, and the IPSS score, and an increase in the lowest SpO_2_ levels postoperatively. However, ESS and QoL-IPSS improvement was only noted in the successful TORS group. There was no significant difference in the baseline AHI between the successful and unsuccessful groups. The successful group showed a greater reduction in AHI three months postoperatively than the unsuccessful group according to the definition. However, the successful group had a higher baseline lowest SpO_2_ than the unsuccessful group (77.14 ± 9.95 (57–92) vs. 71.12 ± 8.33 (47–84), *p* = 0.027). The increment of lowest SpO_2_ in the successful group was marginally significant in comparison to the unsuccessful group (9.00 ± 9.16 vs. 4.11 ± 7.78, *p* = 0.052). The IIEF score significantly improved only in the unsuccessful group. The baseline ED was 36.3% (8/22) in the successful group and 65.3% (17/26) in the unsuccessful group. We further analyzed the baseline ED (IIEF ≤ 21) patients to evaluate the effect of successful TORS for OSA on IIEF improvement. A statistically significant improvement in the IIEF score was still only noted in the unsuccessful group (Table 4).

In the patients with moderate-to-severe LUTS (IPSS ≥ 8) at baseline, QOL-IPSS, storage symptoms, voiding symptoms, and the IPSS score had a significant decrease (*p* < 0.05) postoperatively. Inversely, no obvious changes were found in the patients without moderate-to-severe LUTS (IPSS < 8) at baseline. The patients with ED (IIEF ≤ 21) at baseline were older than the patients without ED (IIEF > 21) at baseline (*p* = 0.027). Both groups had a significant improvement in BW and AHI. In the patients with ED at baseline, IIEF was found to significantly increase from 18.28 ± 2.17 to 20.40 ± 4.08 (*p* = 0.008), but no obvious change was seen in the patients without ED at baseline (*p* = 0.505) (Table 5).

### 3.3. Correlation between BW, IPSS, IIEF, and AHI

**Figure 1:**
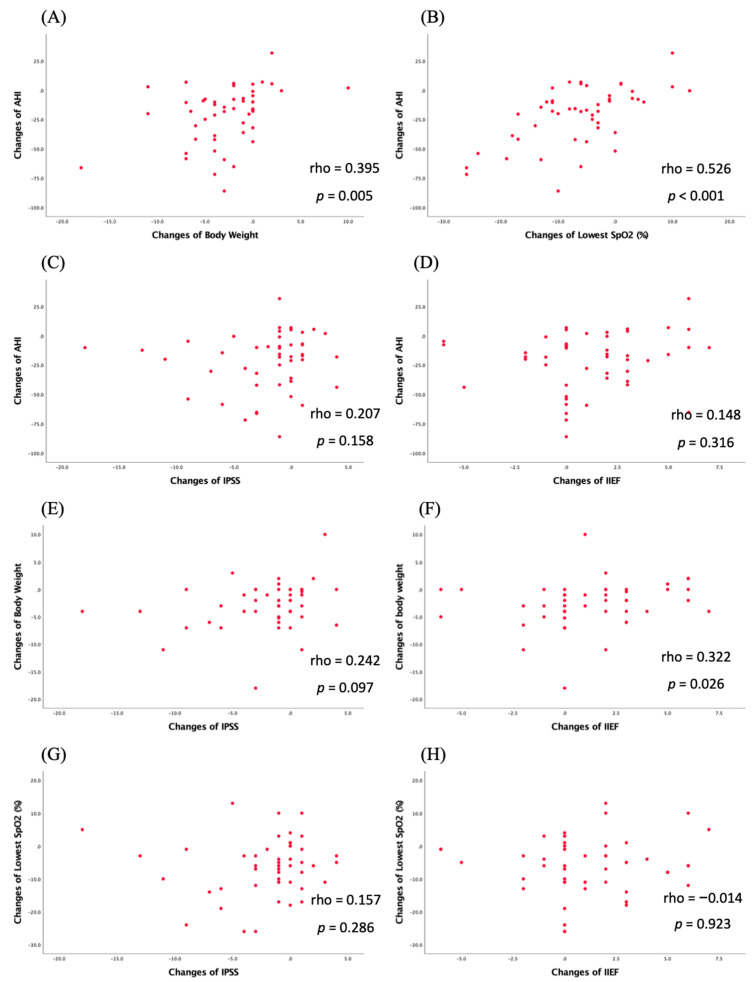
The association between AHI, lowest SpO_2,_ body weight, IPSS, and IIEF. (**A**,**B**) Correlation between the changes in AHI and the changes in BW and the lowest SpO_2_; (**C**,**D**) correlation between changes in AHI and changes in IPSS and IIEF; (**E**,**F**) correlation between the changes in BW and the changes in IPSS and IIEF; (**G**,**H**) correlation between the changes in the lowest SpO_2_ and the changes in IPSS and IIEF.

Our analysis showed that the AHI changes were strongly correlated with the changes in BW and the lowest SpO_2_, respectively (rho = 0.395, *p* = 0.005 and rho = 0.526, *p* < 0.001). The AHI changes had a weak correlation with the changes in IPSS and IIEF. Furthermore, BW loss was found to have a significant correlation with the changes in IIEF (rho = 0.322, *p* = 0.026) in comparison to IPSS (rho = 0.242, *p* = 0.097). The lowest SpO_2_ improvement only had a weak correlation with the changes in IIEF and IPSS (Figure 1). In our study, the changes in AHI, BW, and the lowest SpO_2_ had no significant correlation with IPSS question 7 (nocturia).

### 3.4. Postoperative Adverse Events

All of the enrolled subjects had neither postoperative airway compromise nor acute bleeding requiring treatment or further follow-up. No patients with adverse events warranted surgical intervention and/or readmission. Sore throat with mild swallowing difficulty and mild aspiration were improved and had no impact on QOL three months postoperatively.

## 4. Discussion

In our study, we reviewed 48 consecutive male patients with moderate-to-severe OSA who had undergone TORS. According to Sher’s criteria [21] for OSA surgery, our success rate was 45.8% (22/48). However, both the successful and unsuccessful TORS groups demonstrated improvements in BW, neck circumference, lowest SpO_2_, AHI, and IPSS. The patients with baseline moderate-to-severe LUTS (IPSS ≥ 8) and ED (IIEF ≤ 21) had a significant improvement in IPSS and IIEF, respectively, postoperatively. The reduction in AHI was significantly associated with the changes in BW and the lowest SpO_2_, but not with the changes of in IPSS and IIEF.

Although the plausible pathophysiological pathways linking LUTS and ED have not been clearly elucidated, the possible association has resulted in a new approach to the evaluation and treatment of these conditions [22,23]. Previous research has inferred possible common theories of how both conditions are interrelated, i.e., the nitric oxide synthase/nitric oxide (NOS/NO) theory, the autonomic hyperactivity and metabolic syndrome hypothesis, the Rho-kinase activation/endothelin pathway, and pelvic atherosclerosis [7]. Repetitive hypoxia due to OSA provokes the release of inflammatory cytokines. These cytokines could stimulate cascade inflammatory responses and oxidative stress, then lead to endothelial dysfunction, sympathetic nervous system activation, and metabolic dysregulation [24]. Moreover, several studies have suggested that these inflammatory mechanisms and subsequent pathways might play key roles in linking OSA with LUTS and ED [2,25,26].

Accumulated data have suggested that treatment of ED in OSA patients using either CPAP or oral appliances was promising [27]. Another study pointed out that UPPP treatment could have positive effects on erectile function, but it could also limit aspects related to the ability to cure OSA [20]. TORS offers surgeons improved dexterity and precision, advanced imaging techniques with three-dimensional depth perception, and fewer limitations within the operative field than traditional or laparoscopic surgery. TORS also permits removal of obstructive tissue in the tongue base and the potential to obviate the need for external incisions, pharyngotomy, and tracheotomy [28]. Only one study investigating the effects of TORS-OSA surgery on LUTS and overactive bladder (OAB) has been reported in the literature [29]. Unfortunately, that study did not evaluate ED. Although our enrolled moderate-to-severe OSA patients presented with mild baseline IPSS and IIEF in comparison to previous studies, in addition to OSA-related parameters, such as AHI, ESS, and lowest SpO_2_, the IPSS and IIEF scores were significantly improved three months after TORS-OSA surgery. ED and cardiovascular disorder (CVD) should be considered two different manifestations of the same systemic disorder. The significance of OSA for the development of CVD is widely accepted [30]. ED usually precedes CVD, and its diagnosis and treatment offers a window of opportunity for risk mitigation [31]. Therefore, larger and long-term randomized controlled trials are needed to fully elucidate the clinical practice benefit of the postoperative improvements in IIEF and IPSS.

When we divided the patents into subgroups with a median age cut-off (40 years old), we found significant changes in the OSA parameters in both groups. The IPSS score was also alleviated in these two groups. However, significant IIEF changes were only noted in the older age group. The reason for this may be partially attributed to relatively good baseline IIEF scores (22.68 ± 2.41, score > 21, without ED) of the younger age group. Another explanation was that the degree of ED at baseline showed an inverse correlation with the improvements in ED after treatment [20].

ED, as defined by an IIEF score ≤ 21, was present in 25 patients (25/48, 52%) in our cohort. Our findings are supported by several other studies that reported a high prevalence (30–68%) of ED in patients with OSA [32]. Our ED ratio is lower than the 68.1% reported in Shulz et al. [11] although both studies had a similar AHI (53.1 ± 25.77 vs. 56.0 ± 2.3). The differences in the demographic characteristics, including younger patients (40.6 ± 8.1 vs. 51.5 ± 0.9) and slimmer patients (BMI 28.24 ± 5.38 vs. 34.2 ± 0.6), in our study may have also contributed to this discrepancy. We further classified the patients into two groups according to baseline ED (IIEF ≤ 21) or a no ED group (Table 5). The patients in the baseline ED group were older than their counterparts (43.0 ± 7.4 vs. 37.9 ± 8.2, *p* = 0.027). Both the ED and the no ED groups demonstrated significant improvement in the OSA parameters (AHI, lowest SpO_2_) after surgery, but only the baseline ED group had significant improvement in the IIEF scores. This finding is in agreement with the study conducted by Zheng et al., which showed no significant differences between the ED and no ED groups in terms of the PSG parameters, respiratory disturbance index, and the lowest SpO_2_ value [33]. They postulated that the severity of OSA was not significantly associated with ED, according to their correlation analyses or the multivariate logistic regression analysis.

In our study, we allocated the patients into two subgroups on the basis of Sher’s success criteria: those with more than a 50% reduction in AHI and those with a reduction in AHI of less than 20 per hour. Our success rate of TORS for OSA was 45.8%. Several studies have reported that the success rate of UPPP ranges from 35% to 70% in non-selected OSA patients [34]. Both groups had significant improvements in BW, neck circumference, IPSS, lowest SpO_2_, and AHI. However, the successful TORS for OSA was found to have a better outcome in terms of lowest SpO_2_ increment and AHI alleviation than the unsuccessful group (Table 4). It is suggested that the increase in the pulse oxygen decline rate can lead to the aggravation of daytime sleepiness in patients with severe OSA. After apnea, the pulse oxygen decline rate increases; when the body is hypoxic, the oxidative stress is enhanced and the inflammatory level is increased. The inflammatory cytokine accumulation compromises the endothelium of the brain, spinal cord, and pelvic organs, which can lead to sleepiness symptoms and LUTS [35]. These theories are consistent with the reason why a significant reduction in ESS and IPSS was noted in the successful TORS for OSA group in our study. No significant and marginally significant improvement in ESS and IPSS was seen in the unsuccessful group. Unexpectedly, significant IIEF enhancement was only noted in the unsuccessful group, even with the subgroup analysis of baseline ED. We speculate that there are several reasons for these findings. Although a decrease in AHI was only one criterion for successful TORS-OSA outcome, a correlation was found between AHI reduction and IPSS alleviation and IIEF enhancement (Figure 1C,D). Our correlation analysis showed that the changes in BW were correlated with the changes in IIEF scores (rho = 0.322, *p* = 0.026) (Figure 1F). Weight loss in men with OSA associated with a regain of normal erectile function had also been reported [36]. Moreover, the severity of ED according to the IIEF score has been linked to nocturnal hypoxemia but not to apnea frequency [33]. Some researchers have raised the idea that the lowest SpO_2_, not AHI, is a significant predictor of the presence of ED in OSA [37]. Lastly, in our study there were 17 (17/26, 65.3%) patients with baseline ED in the unsuccessful TORS for OSA group but only 8 (8/22, 36.3%) patients with baseline ED in the successful group (*p* = 0.045), which may skew the findings. There is also growing evidence to support the idea of evaluating the surgical outcome directly by continuously documenting first-person experiences after surgery instead of using AHI as a sole surrogate [38]. The pathophysiology of ED in OSA is multifactorial and is even more complicated than that of LUTS.

In recent evidence, Irer et al. reported men aged ≤ 50 years had a significantly improved IPSS score (from 8.4 ± 6.3 to 2.8 ± 2.5) and IIEF score (from 17.0 ± 4.9 to 24.2 ± 4.8) after CPAP treatment for mild-to-severe OSA for three months [9]. Shin et al. reported an IIEF increase from 15.6 ± 6.8 to 17.8 ± 5.5 in OSA patients six months after UPPP [20]. As a general rule, nocturia is an important index for OSA treatment. Other studies have reported that OSA patients who underwent UPPP had significant improvement in nocturia and their IPSS scores three months postoperatively, especially in the successful group [5,12]. Baseline AHI was reported to be significantly correlated with baseline nocturia [5]. However, no significant association was found between AHI reduction and improvement of nocturia in both the successful and unsuccessful groups [5]. Our patients were found to have significant IPSS improvement (*p* = 0.001), but only a marginally significant change in nocturia (1.06 ± 0.95 to 0.83 ± 0.63). Most of our patients (37/48) had nocturia less than twice, which made the nocturia analysis uncertain.

Nocturnal gastroesophageal reflux disease (GERD) is a common symptom observed in OSA patients due to similar shared risk factors, such as obesity. In our study, 26 patients (54.2%) had GERD symptoms, but no questionnaire or other examination was used to evaluate improvement of GERD after TORS. Previous studies indicated that up to 73% of OSA patients also had GERD. The causal link between OSA and GERD is still unknown; however, lifestyle modifications for BW loss or CPAP can effectively improve both sleep quality and gastroesophageal reflux [39,40,41]. A meta-analysis revealed that CPAP treatment significantly reduces the number of reflux events, the reflux disease questionnaire score, and the reflux symptom index [42]. The effects of TORS for OSA on gastroesophageal reflux need to be further investigated.

Our study has several limitations. First, it was conducted on a small population and not based on a power calculation. This precluded us from using more robust statistical methods, such as multivariate analysis. However, all the patients that underwent TORS for OSA had an OSA status of moderate-to-severe, and all the surgeries were performed by the same otolaryngologist with experience in performing TORS on at least 100 cases. Second, the patients served as their own control before and after TORS for OSA; the study did not include patients treated with CPAP or UPPP as a control group. However, the aim of our study was to evaluate if using TORS to treat OSA would affect ED and LUTS. The comparison of TORS, CPAP, and traditional UPPP may lead to loss of focus. Third, our data only indicated the severity of ED or LUTS without a timeframe for these conditions. Thus, it was not possible to determine the influence of ED and LUTS duration on the recovery effect of TORS for OSA. Fourth, ED and LUTS were only gauged by questionnaires and with a short follow-up. The effect of TORS for OSA on ED and LUTS may need a longer follow-up time and a urodynamic study or related hormone survey for a more objective evaluation. Fifth, most of our patients did not present with nocturia ≥ 2 times/per night. This deprived us of the possibility of analyzing the improvement of nocturia after surgery. Lastly, TORS for OSA is only available in selected centers, and not every patient can afford the expense of this novel operation. Nevertheless, our report was the first prospective study to evaluate the effect of TORS for OSA on both ED and LUTS.

## 5. Conclusions

In conclusion, TORS for men with OSA can significantly improve the PSG parameters, sleep-related questionnaire scores, and QOL; it can also alleviate ED and LUTS. AHI reduction is not a crucial factor for improving ED and LUTS after TORS for OSA; this is especially the case for ED.

## Figures and Tables

**Table 1:** Baseline and postoperative characteristics (total N = 48).

	Pre-Surgery	Post-Surgery	*p* Value
**Age**	40.6 ± 8.1 (21–55)		
**Severity of OSA**			
Moderate (15 ≤ AHI < 30)	11 (22.9%)		
Severe (AHI ≥ 30)	37 (77.1%)		
**Past history**			
Hypertension	10 (20.8%)		
Hepatitis B	2 (4.1%)		
Hyperlipidemia	3 (6.2%)		
Gout	4 (8.3%)		
GERD	26 (54.2%)		
CAD	1 (2.1%)		
Smoking	12 (25.0%)		
**Prior treatment for OSA**			
CPAP	24 (50%)		
Oral appliance	6 (12.5%)		
Unknown	4 (8.3%)		
Refuse prior treatment	14 (29.2%)		
**BW**	85.16 ± 17.36 (63.0–172.0)	82.28 ± 15.73 (63.0–154.0)	<0.001
**BMI**	28.24 ± 5.38 (20.20–53.09)	27.28 ± 4.90 (19.88–47.53)	<0.001
**ESS**	8.15 ± 4.73 (0–19)	6.19 ± 3.42 (0–15)	0.005
**Neck circumference**	39.65 ± 2.36 (36.0–47.2)	38.88 ± 2.65 (33.0–45.5)	0.001
**Lowest SpO_2_ (%)**	73.88 ± 9.51 (47–92)	80.23 ± 9.45 (51–95)	<0.001
**AHI**	53.10 ± 25.77 (15.1–110.2)	31.66 ± 20.34 (0.90–73.3)	<0.001

GERD = Gastroesophageal Reflux Disease, CAD = Coronary Artery Disease, CPAP = Continuous Positive Airway Pressure, BW = Body Weight, BMI = Body Mass Index, ESS = the Epworth Sleepiness Scale, SpO_2_ = oxygen saturation, AHI = Apnea–Hypopnea Index.

**Table 2:** Postoperative changes in the IPSS and IIEF scores (N = 48).

	Pre-Surgery	Post-Surgery	*p* Value
**IPSS questions**			
Incomplete Emptying	0.90 ± 1.35	0.50 ± 0.98	0.021
Frequency	1.00 ± 1.28	0.69 ± 0.68	0.034
Intermittency	0.52 ± 1.09	0.21 ± 0.41	0.046
Urgency	0.75 ± 1.17	0.38 ± 0.70	0.030
Weak stream	0.54 ± 1.09	0.23 ± 0.42	0.024
Straining	0.29 ± 0.71	0.15 ± 0.35	0.197
Nocturia	1.06 ± 0.95	0.83 ± 0.63	0.047
**Storage**	2.81 ± 2.49	1.90 ± 1.51	0.002
**Voiding**	2.25 ± 3.52	1.08 ± 1.52	0.011
**IPSS**	5.06 ± 5.42	2.98 ± 2.71	0.001
**QoL-IPSS**	1.60 ± 1.42	1.08 ± 1.06	0.005
**IIEF**	20.98 ± 3.32	22.17 ± 3.60	0.007

IPSS = International Prostate Symptom Score, QoL-IPSS = Quality of Life in IPSS, IIEF = International Index of Erectile Function. Storage symptoms including frequency, urgency and nocturia; Voiding symptoms including incomplete emptying, intermittency, weak stream, and straining to void.

**Table 3:** Postoperative changes in the younger and older age subgroups.

	Younger (N = 22, Age < 40)	Older (N = 26, Age ≥ 40)	*p* Value
**Age**	33.0 ± 4.7 (21–39)	47.0 ± 3.7 (40–55)	<0.001
**BW**			
Pre-Surgery	88.68 ± 23.11 (64.0–172.0)	82.18 ± 9.88 (63.0–101.0)	0.200
Post-Surgery	86.04 ± 20.32 (63.0–154)	79.10 ± 9.77 (63.0–100.0)	0.154
Body weight loss	2.63 ± 5.08 (*p* = 0.024)	3.08 ± 3.46 (*p* < 0.001)	0.722
**ESS**			
Pre-Surgery	8.50 ± 5.09 (1–19)	7.85 ± 4.47 (0–18)	0.638
Post-Surgery	6.55 ± 3.06 (2–15)	5.88 ± 3.73 (0–14)	0.511
Reduced ESS	1.95 ± 5.18 (*p* = 0.092)	1.96 ± 4.17 (*p* = 0.024)	0.996
**Neck circumference**			
Pre-Surgery	39.33 ± 2.39 (36.0–47.2)	39.91 ± 2.34 (36.0–45.5)	0.403
Post-Surgery	38.72 ± 2.80 (33.0–45.5)	39.01 ± 2.57 (34.0–45.0)	0.704
Reduced circumference	0.61 ± 1.36 (*p* = 0.047)	0.89 ± 1.54 (*p* = 0.007)	0.510
**Lowest SpO_2_ (%)**			
Pre-Surgery	71.59 ± 10.48 (47–90)	75.81 ± 8.32 (54–92)	0.127
Post-Surgery	79.91 ± 9.74 (57–92)	80.50 ± 9.39 (51–95)	0.832
Increased Lowest SpO_2_	8.31 ± 9.27 (*p* < 0.001)	4.69 ± 7.99 (*p* = 0.006)	0.152
**AHI**			
Pre-Surgery	56.15 ± 27.94 (15.1–109.7)	50.51 ± 24.02 (15.1–110.2)	0.456
Post-Surgery	32.10 ± 21.75 (0.9–73.3)	31.30 ±19.49 (4.0–66.3)	0.894
Reduced AHI	24.05 ± 30.46 (*p* = 0.001)	19.21 ± 18.71 (*p* < 0.001)	0.521
**QoL-IPSS**			
Pre-Surgery	1.36 ± 1.39 (0–5)	1.81 ± 1.44 (0–4)	0.287
Post-Surgery	0.95 ± 1.13 (0–4)	1.19 ± 1.02 (0–3)	0.448
Reduced QoL-IPSS	0.40 ± 1.00 (*p* = 0.071)	0.61 ± 1.38 (*p* = 0.033)	0.565
**IPSS**			
Pre-Surgery	4.14 ± 4.33 (0–18)	5.85 ± 6.16 (0–24)	0.267
Post-Surgery	2.59 ± 2.75 (0–10)	3.31 ± 2.69 (0–9)	0.368
Reduced IPSS	1.54 ± 2.48 (*p* = 0.008)	2.53 ± 5.35 (*p* = 0.023)	0.404
**IIEF**			
Pre-Surgery	22.68 ± 2.41 (17–25)	19.54 ± 3.33 (12–25)	0.001
Post-Surgery	23.45 ± 2.73 (15–25)	21.08 ± 3.92 (10–25)	0.021
Increased IIEF	0.77 ± 2.36 (*p* = 0.141)	1.53 ± 3.30 (*p* = 0.025)	0.369

BW = body weight, ESS = The Epworth Sleepiness Scale, SpO_2_ = oxygen saturation, AHI = Apnea–Hypopnea Index, IPSS = International Prostate Symptom Score, QoL-IPSS = Quality of Life in IPSS, IIEF = International Index of Erectile Function, LUTS = lower urinary tract symptoms, ED = erectile dysfunction.

**Table 4:** Postoperative changes in the successful TORS and unsuccessful TORS subgroups.

	Successful Group (N = 22)	Unsuccessful Group (N = 26)	*p* Value
**Age**	39.3 ± 7.2 (25–48)	41.6 ± 8.8 (21–55)	0.322
**BW**			
Pre-Surgery	87.90 ± 23.19 (64.0–172.0)	82.84 ± 10.13 (63.0–102.0)	0.319
Post-Surgery	83.99 ± 20.32 (63.0–154.0)	80.84 ± 10.67 (63.0–105.0)	0.496
Body weight loss	3.91 ± 3.81 (*p* < 0.001)	1.99 ± 4.45 (*p* = 0.031)	0.119
**ESS**			
Pre-Surgery	7.95 ± 4.98 (1–19)	8.31 ± 4.60 (0–18)	0.800
Post-Surgery	5.50 ± 3.14 (1–15)	6.77 ± 3.60 (0–14)	0.204
Reduced ESS	2.45 ± 4.60 (*p* = 0.021)	1.53 ± 4.66 (*p* = 0.105)	0.499
**Neck circumference**			
Pre-Surgery	39.30 ± 2.44 (36.0–47.2)	39.93 ± 2.30 (36.0–45.5)	0.363
Post-Surgery	38.60 ± 2.86 (33.0–45.5)	39.12 ± 2.49 (34.5–45.0)	0.503
Reduced circumference	0.70 ± 1.26 (*p* = 0.016)	0.81 ± 1.63 (*p* = 0.017)	0.805
**Lowest SpO_2_ (%)**			
Pre-Surgery	77.14 ± 9.95 (57–92)	71.12 ± 8.33 (47–84)	0.027
Post-Surgery	86.14 ± 5.11 (73–95)	75.23 ± 9.45 (51–88)	0.001
Increased Lowest SpO_2_	9.00 ± 9.16 (*p* < 0.001)	4.11 ± 7.78 (*p* = 0.012)	0.052
**AHI**			
Pre-Surgery	53.53 ± 30.17 (15.1–109.7)	52.74 ± 21.99 (15.1–110.2)	0.917
Post-Surgery	16.87 ±10.87 (0.9–42.1)	44.18 ± 17.97 (20.3–73.3)	<0.001
Reduced AHI	36.65 ± 24.93 (*p* < 0.001)	8.55 ± 15.56 (*p* = 0.010)	<0.001
**QoL-IPSS**			
Pre-Surgery	1.64 ± 1.59 (0–5)	1.58 ± 1.30 (0–4)	0.887
Post-Surgery	0.82 ± 1.09 (0–4)	1.31 ± 1.01 (0–3)	0.115
Reduced QoL-IPSS	0.81 ± 1.29 (*p* = 0.007)	0.26 ± 1.11 (*p* = 0.230)	0.122
**IPSS**			
Pre-Surgery	4.41 ± 4.15 (0–16)	5.62 ± 6.32 (0–24)	0.448
Post-Surgery	2.23 ± 2.11 (0–10)	3.62 ± 3.03 (0–9)	0.070
Reduced IPSS	2.18 ± 3.43 (*p* = 0.007)	2.00 ± 4.94 (*p* = 0.049)	0.885
**Baseline IPSS ≥ 8 (LUTS)**	n = 4 (4/22, 18.1%)	n = 7 (7/26, 26.9%)	0.473 *
Pre-Surgery	11.75 ± 3.50 (8–16)	14.71 ± 4.88 (10–24)	0.318
Post-Surgery	4.00 ± 4.08 (1–10)	6.14 ± 1.95 (4–9)	0.260
Reduced IPSS	7.75 ± 4.27 (*p* = 0.036)	8.57 ± 4.79 (*p* = 0.003)	0.783
**IIEF**			
Pre-Surgery	21.82 ± 2.98 (16–25)	20.27 ± 3.48 (12–25)	0.108
Post-Surgery	22.64 ± 3.47 (11–25)	21.77 ± 3.73 (10–25)	0.412
Increased IIEF	0.81 ± 2.38 (*p* = 0.122)	1.50 ± 3.30 (*p* = 0.029)	0.424
**Baseline IIEF ≤ 21 (ED)**	n = 8 (8/22, 36.3%)	n = 17 (17/26, 65.3%)	0.045 *
Pre-Surgery	18.38 ± 1.76 (16–21)	18.24 ± 2.38 (12–21)	0.884
Post-Surgery	19.88.38 ± 4.42 (11–24)	20.65 ± 4.03 (10–25)	0.669
Increased IIEF	1.50 ± 3.70 (*p* = 0.290)	2.41 ± 3.69 (*p* = 0.016)	0.571

BW = body weight, ESS = The Epworth Sleepiness Scale, SpO_2_ = oxygen saturation, AHI = Apnea–Hypopnea Index, IPSS = International Prostate Symptom Score, QoL-IPSS = Quality of Life in IPSS, IIEF = International Index of Erectile Function, LUTS = lower urinary tract symptoms, ED = erectile dysfunction, Successful TORS: the reduction of at least 50% in preoperative AHI or a postoperative AHI < 20 per hour, * chi-squared test.

**Table 5:** Postoperative changes in the subgroup of patients with ED at baseline (IIEF ≤ 21).

	ED (N = 25)	No ED (N = 23)	*p* Value
**Age**	43.0 ± 7.4 (27–55)	37.9 ± 8.2 (21–50)	0.027
**BW**			
Pre-Surgery	84.99 ± 20.76 (63.0–172.0)	85.34 ± 13.17 (64.0–113.0)	0.945
Post-Surgery	82.92 ± 17.76 (63.0–154)	81.60 ± 13.56 (63.0–110.0)	0.775
Body weight loss	2.07 ± 4.16 (*p* = 0.020)	3.74 ± 4.24 (*p* < 0.001)	0.175
**Neck circumference**			
Pre-Surgery	39.88 ± 2.61 (36.0–47.2)	39.40 ± 2.08 (36.0–45.5)	0.488
Post-Surgery	39.00 ± 2.81 (34.0–45.5)	38.74 ± 2.53 (33.0–43.3)	0.739
Reduced circumference	0.87 ± 1.41 (*p* = 0.005)	0.65 ± 1.53 (*p* = 0.053)	0.608
**Lowest SpO_2_ (%)**			
Pre-Surgery	74.60 ± 8.47 (54–88)	73.09 ± 10.66 (47–92)	0.587
Post-Surgery	79.96 ± 8.51 (51–91)	80.52 ± 10.57 (57–95)	0.840
Increased Lowest SpO_2_	5.36 ± 8.40 (*p* = 0.004)	7.43 ± 9.08 (*p* = 0.001)	0.415
**AHI**			
Pre-Surgery	51.06 ± 24.97 (15.1–110.2)	55.32 ± 26.99 (15.1–109.7)	0.573
Post-Surgery	32.17 ± 19.30 (4.8–68.8)	31.12 ± 21.83 (0.9–73.3)	0.860
Reduced AHI	18.89 ± 22.72 (*p* < 0.001)	24.20 ± 26.78 (*p* < 0.001)	0.462
**IPSS**			
Pre-Surgery	6.04 ± 5.89 (1–24)	4.00 ± 4.74 (0–16)	0.196
Post-Surgery	4.16 ± 2.99 (0–10)	1.70 ± 1.63 (0–5)	0.001
Reduced IPSS	1.88 ± 4.54 (*p* = 0.049)	2.30 ± 4.05 (*p* = 0.012)	0.735
**IIEF**			
Pre-Surgery	18.28 ± 2.17 (12–21)	23.91 ± 1.08 (22–25)	<0.001
Post-Surgery	20.40 ± 4.08 (10–25)	24.09 ± 1.47 (20–25)	<0.001
Increased IIEF	2.12 ± 3.64 (*p* = 0.008)	0.17 ± 1.23 (*p* = 0.505)	0.017

BW = body weight, SpO_2_ = oxygen saturation, AHI = Apnea–Hypopnea Index, IPSS = International Prostate Symptom Score, IIEF = International Index of Erectile Function.

## Data Availability

The data presented in this study are available on request from the corresponding author upon reasonable request.
